# Host Cytokine Responses Induced after Overnight Stimulation with Novel *M. tuberculosis* Infection Phase-Dependent Antigens Show Promise as Diagnostic Candidates for TB Disease

**DOI:** 10.1371/journal.pone.0102584

**Published:** 2014-07-15

**Authors:** Paulin N. Essone, Novel N. Chegou, Andre G. Loxton, Kim Stanley, Magdalena Kriel, Gian van der Spuy, Kees L. Franken, Tom H. Ottenhoff, Gerhard Walzl

**Affiliations:** 1 DST/NRF Centre of Excellence for Biomedical Tuberculosis Research and MRC Centre for Tuberculosis Research, Division of Molecular Biology and Human Genetics, Department of Biomedical Sciences, Faculty of Medicine and Health Sciences, Stellenbosch University, Tygerberg, South Africa; 2 Department of Infectious Diseases, Leiden University Medical Centre, Leiden, The Netherlands; University of Palermo, Italy

## Abstract

**Background:**

We previously identified Mycobacterium *tuberculosis* (*M.tb*) antigen-induced host markers that showed promise as TB diagnostic candidates in 7-day whole blood culture supernatants. The aim of the present study was to evaluate the utility of these markers further, and cross-compare results with short-term antigen stimulated and unstimulated culture supernatants.

**Methods:**

We recruited 15 culture confirmed TB cases and 15 non-TB cases from a high-TB endemic community in Cape Town, South Africa into a pilot case-control study from an on-going larger study. Blood samples collected from study participants were stimulated with 4 *M.tb* antigens that were previously identified as promising (ESAT6/CFP10 (early secreted), Rv2029c (latency), Rv2032 (latency) and Rv2389c (rpf)) in a 7-day or overnight culture assay. Supernatants were also collected form the standard QuantiFERON In Tube (QFT-IT) test. The levels of 26 host markers were evaluated in the three culture supernatants using the Luminex platform.

**Results:**

The unstimulated levels of CRP, Serum amyloid P (SAP) and serum amyloid A (SAA) and ESAT-6/CFP-10 specific IP-10 and SAA were amongst the best discriminatory markers in all 3 assays, ascertaining TB with AUC of 72–84%. Four-marker models accurately classified up to 92%, 100% and 100% of study participants in the overnight, 7-day and Quantiferon culture supernatants, respectively, after leave-one-out cross validation.

**Conclusion:**

Unstimulated and antigen-specific levels of CRP, SAA, IP-10, MMP-2 and sCD40L hold promise as diagnostic candidates for TB disease in short-term stimulation assays. Larger studies are required to validate these findings but the data suggest that antigen-specific cytokine production and in particular mutimarker biosignatures might contribute to future diagnostic strategies.

## Background

The diagnosis of tuberculosis (TB) disease remains a challenge in resource-constrained settings [1]. The sputum based tests widely used to diagnose active TB have many limitations: staining for acid fast bacilli, the most widely used test has poor sensitivity [2], [3]. Sputum culture remains the gold standard method for TB diagnosis but fails to deliver results in a time effective manner [4]. The automated real-time sputum processing molecular beacon assay, XpertMTB/RIF assay (Cepheid Inc., CA, USA) yields results within 2 hours, with high sensitivity and specificity (98–100%) in smear positive but moderate sensitivity (68–72%) in smear negative TB cases [5], [6]. Cost effectiveness of the GeneXpert test remains one of the major impediments to the large-scale roll-out of the test in high burden but resource-constrained settings [7], [5]. Alternative methods such as serological, urine based [8], [9], and other immunological tests have been investigated but these tests do not yield consistent results. The use of the available commercial serological tests was subsequently discouraged by the WHO [1].

Interferon gamma (IFN-γ) release assays (IGRAs) have added a new dimension to the immune-based diagnosis of TB, as these tests are highly specific and accurate in the diagnosis of *Mycobacterium tuberculosis (M.tb)* infection, especially when compared to the skin test [10]. However, IGRAs do not differentiate between active TB disease and latent infection and although useful in low incidence settings, they are only used for research purposes in high burden areas [1], [10], [11]. Alternative *M.tb* specific antigens [12]–[13], and alternative host markers other than IFN-γ in *M.tb*-specific antigen-stimulated whole blood culture assays have been investigated for TB diagnostic purposes [14], [15]. In one of these studies [15] we showed that the measurement of many host markers (including interferon-inducible protein (IP)-10, tumour necrosis factor (TNF)-α, IFN-γ, interleukin (IL)-12p40, transforming growth factor (TGF)-α, vascular endothelial growth factor (VEGF) and RANTES (CCL5)) upon stimulation with novel *M.tb* infection phase-dependent antigens (including Rv0081, Rv2032, Rv1737c, Rv2389c and Rv0867c) in culture supernatants showed potential in the diagnosis of TB disease [15]. However, in those studies we employed a long term (7-day) whole blood culture method, which might not be very suitable for diagnostic purposes. In the current study, we investigated whether these novel antigens [15] can also induce host markers after stimulation in short-term (overnight) assays and whether the diagnostic potential observed after 7-day WBA is maintained. We show that these antigens induce the same and other host markers in overnight culture assays, and maintain their diagnostic potential.

## Materials and Methods

### Study participants

The study was performed as a pilot study to a larger study, the EDCTP-funded African-European Tuberculosis Consortium (AE-TBC) project, that was conducted in 7 African and 5 European institutions (www.ae-tbc.eu). Participants visiting a clinic in one of the African sites, the Ravensmead/Uitsig community clinic in the Western Cape Province of South Africa, with symptoms suspicious of TB and on whom the clinician was willing to consider for further TB tests were enrolled into the study. All study participants underwent a thorough clinical workup including chest x-rays and HIV testing using a rapid test (Abott, Germany). Blood and sputum samples, along with other samples needed for routine clinical investigations were collected from all study participants. Sputum samples were cultured using the MGIT method (BD Biosciences). Isolation of *M.tb* complex organisms was confirmed in all positive cultures by means of an *M.tb* complex specific PCR [3]. Participants were eligible for the study if they were ≥18 years old, had no previous history of TB, were not pregnant, were not involved in a drug or vaccine trial and if they had no other known chronic diseases like diabetes mellitus. According to the TB test outcomes, participants were divided into two groups; confirmed TB cases (the TB disease group in this manuscript) and alternative pulmonary disease (referred to as the “Non-TB” cases in this study). None of the participants in the non-TB group was treated for TB by the national TB control program during the 6 month follow up period of the study. All participants provided written informed consent for participation in the study including for HIV testing, storage and use of the samples for immunological biomarker discovery purposes. Ethical approval for the study was obtained from the Human Research Ethics Committee of the University of Stellenbosch (N10/08/274).

### Whole blood culture assays (WBA)

About 10 ml of whole blood was collected from all study participants into heparinized tubes for overnight and long term (7-day) WBAs. Furthermore, 3 ml of blood was collected directly into QFT-IT tubes (Qiagen, Germany).

Four recombinant proteins, generated at Leiden University Medical Centre, The Netherlands, (Rv2029c, Rv2032, Rv2389c and ESAT6/CFP10 fusion protein), previously described in [12, [15] [16], were available for evaluation in this study. All the antigens were reconstituted and evaluated at a final concentration of 10 µg/ml in an overnight and 7-day WBA as follows:

The 7-day WBA was performed as previously described [12]. Briefly, lyophilized antigens were reconstituted in sterile 1x PBS and diluted to a concentration of 20 µg/ml with RPMI 1640 containing L-glutamine (Sigma Aldrich, Steinheim, Germany). The diluted antigens (100 µl at 20 µg/ml) as well as the medium (unstimulated control), were then seeded into 96-well plates in triplicates after which plates were frozen at −80°C until the day of WBA. On the day of WBA, pre-frozen antigen plates were allowed to thaw after which whole blood was diluted 1 in 5 in pre-warmed (37°C) RPMI1640 medium containing glutamine, and then 100 µl of the diluted blood added into each well containing the antigens or control. The plate was then incubated at 37°C at 5% CO_2_ until day 7 after which supernatants were harvested, aliquoted and stored at −80°C until testing using the Luminex platform (described below).

For the overnight WBA, the reconstituted antigens (in sterile 1x PBS) were diluted to a concentration of 100 µg/ml using sterile 1x PBS. Each diluted antigen (at 100 µg/ml) was then aliquoted in 100 µl amounts and frozen at −80°C in micro centrifuge tubes (Eppendorf Germany). On the day of WBA, an aliquot of each antigen (100 µl at 100 µg/ml) was thawed and added to 1 ml of undiluted whole blood in 24-well tissue culture plates (Corning Costar, Sigma). 100 µl of sterile 1xPBS was added to a single well of the 24-well plate for each participant and evaluated as the unstimulated control. After mixing, plates were incubated for 20 to 24 hours at 37°C in a 5% CO_2_ atmosphere. Supernatants were harvested, aliquoted and frozen at −80°C until evaluated using the Luminex platform.

The QFT-IT assay was performed according to the manufacturer’s instructions as previously described [14]. Briefly, after overnight incubation of the blood in the QFT-IT tubes, supernatants were harvested, aliquoted and frozen at −80°C. Aliquots of supernatants from all participants were used for IFN-γ ELISA using kits supplied by the manufacturer, and interpreted for *M.tb* infection using the manufacturer’s recommended criteria (Qiagen, Germany). Other aliquots were used for the Luminex assay.

### Luminex multiplex immunoassay

A total of 26 host markers, namely epithermal growth factor (EGF), fractalkine, IP-10, monocyte chemotactic protein (MCP)-1, macrophage inflammatory protein (MIP)-1α, MIP-1β, soluble CD40 ligand (sCD40L), TGF-α, TNF-α, VEGF, matrix metalloproteinase (MMP)-2, MMP-9, RANTES, C-reactive protein (CRP), serum amyloid A (SAA), serum amyloid P (SAP), Interferon (IFN)-α2, IFN-γ, IL-1α, IL-12p40, IL-15, IL-17, IL-4, IL-10, IL-1β and IL-12p70, were evaluated in overnight, 7-day, and QFT-IT culture supernatants from the same study participants, using customized Milliplex kits (Merck Millipore, St. Charles, Missouri, USA). Assays were performed on the Bio-Plex platform (Bio-Rad Laboratories, Hercules, USA) according to the instructions of the kit manufacturer (Merck Millipore). Prior to assaying, samples for the detection of CRP, SAA and SAP were diluted 1 in 8000 using the assay buffer provided in the kit, following optimization experiments. To enable the accurate detection of all the other host markers evaluated, the overnight WBA and QFT-IT culture supernatants were diluted 1in 2 using the kit serum matrix as previously described [12], whereas the 7-day WBA supernatants were tested neat (undiluted) [15]. Samples were evaluated in a blinded manner. All analyte levels in the quality control reagents provided by the kit manufacturer were within the expected ranges. The values obtained for all host markers were automatically corrected for the dilution by the software used for bead acquisition and analysis of median fluorescence intensity (Bio-Plex Manager Software, version 4.1.1).

### Statistical analysis

Comparison between groups (for example TB vs. no TB) was done using the Mann Whitney U test for non-parametric data analysis. The accuracy of all antigen-induced host markers for the diagnosis of TB disease was estimated by performing receiver operator characteristics (ROC) curve analysis. Optimal cut-off values were selected based on the Youden’s index, defined as the largest difference between the sensitivity and 1-specificity, taken over all points on the ROC curve [17]. The predictive abilities of combinations of analytes for TB disease were investigated by performing best subsets general discriminant analysis (GDA), with leave-one-out cross validation as previously described [12]. Data were analysed using the Statistica software (Statsoft, Ohio, USA) and GraphPad prism, version 5.0 (GraphPad Software, San Diego, CA, USA).

## Results

### Study participants

Of the 30 participants enrolled into this study, 6 (25%) were males. The mean age of all study participants was 30.0±12.7 years. All the 15 study participants with TB disease had sputum culture and *M.tb-*PCR-confirmed pulmonary TB. Of all the 27 participants in whom QFT-IT testing was done (13 TB cases and 14 non-TB cases), 19 (70.4%) were positive (12 TB cases and 7 non-TB cases) using the manufacturer’s recommended cut-off value (≥0.35IU/ml). The clinical and demographic characteristics of all study participants are shown in table 1.

**Table 1:** Clinical and demographic characteristics of study participants.

	All	Pulmonary TB	No TB
**Number of participants(n)**	30	15	15
**Mean age, years (SD)**	29.9(± 12.5)	26(± 14.2)	33.5(± 10.2)
**Male/female ratio**	6/24	4/11	2/13
**HIV status, (positive/negative)**	3/30	0/15	3/15
**Participants with QFT-IT test results**	27	13	14
**QFT-IT positive, n (%)**	19(70.4)	12(92.3)	7(50)

### Utility of host markers detected in 7-day antigen-stimulated culture supernatants in the diagnosis of TB disease

The unstimulated or antigen-specific levels of 8 of the 26 host markers evaluated (fractalkine, IFN-α2, SAA, IP-10, EGF, IFN-γ, MMP-2, MMP-9) were significantly different (p<0.05) between the TB cases and controls (50% of whom were QFT-IT positive) either in the 7-day unstimulated supernatants, or following 7-day stimulation with at least one of the four antigens evaluated.

The unstimulated levels of fractalkine and SAA were significantly higher in TB cases whereas the unstimulated levels of IFN-α2 and MMP-2 were higher in the non TB cases (table 2).

**Table 2:** Diagnostic potential of markers detected in 7-day culture supernatants for TB disease.

Antigen	Host Marker	TB	No TB	P- value	AUC % (95% CI)	Sensitivity % (95% CI)	Specificity % (95% CI)	Cut off value
**ESAT6/ CFP10**	EGF	6.2 (0–13)	0 (0–5)	0.003	80 (64–96)	67 (38–88)	80 (52–96)	1.470
**ESAT6/ CFP10**	IP-10	4015 (334–15943)	1664 (0–9999)	0.05	71 (53–90)	73 (45–92)	53 (27–79)	1675
**ESAT6/ CFP10**	MMP–9	77644 33259–205268	32611 (0–272113)	0.006	82 (62–98)	87 (60–98)	80 (52–96)	61832
**Rv2029c**	MMP–9	83925 23153–234362	36556 (0–120001)	0.014	77 (60–94)	71 (42–92)	60 (52–96)	68176
**Rv2032**	EGF	8 (0–25)	5 (0–23)	0.023	74 (56–92)	67 (38–88)	60 (60–84)	4.260
**Rv2389c**	EGF	10 (0–27)	1 (0–37)	0.023	74 (56–94)	73 (45–92)	79 (49–95)	8.730
**Rv2389c**	MMP–9	69513 37074–96009	17719 (382–83527)	0.0004	89 (76–100)	93 (68–100)	79 (49–95)	50285
**Unstimulated**	Fractalkine	33 (15–33)	4 (2–432)	0.05	71 (51–91)	100 (78–100)	53 (27–79)	9.455
**Unstimulated**	IFN-α2	2 (2–6)	10 (2–19)	0.038	72 (53–91)	87 (60–98)	60 (32–83)	7.445
**Unstimulated**	MMP-2	251 (112–40475)	5805 (112–66578)	0.023	74 (55–93)	80 (52–96)	73 (45–92)	4359
**Unstimulated**	SAA	22661 (0–90178)	4736 (560–34274)	0.041	72 (53–92)	73 (45–92)	69 (39–91)	630.5

Median levels (and ranges in parenthesis) of analytes detected in 7-day whole blood culture supernatants and accuracy in the diagnosis of TB disease are shown. P-values were calculated using the Mann Whitney U test. Only analytes with AUC≥70% are shown. Cut-off values, sensitivity and specificity were selected based on the Youden’s index. AUC = Area under the receiver operator characteristics curve. 95% CI = 95% confidence interval. All analyte levels are in pg/ml except for SAA (ng/ml). The levels shown for the the different antigens were corrected for background by subtraction of the unstimulated levels.

**Figure 1:**
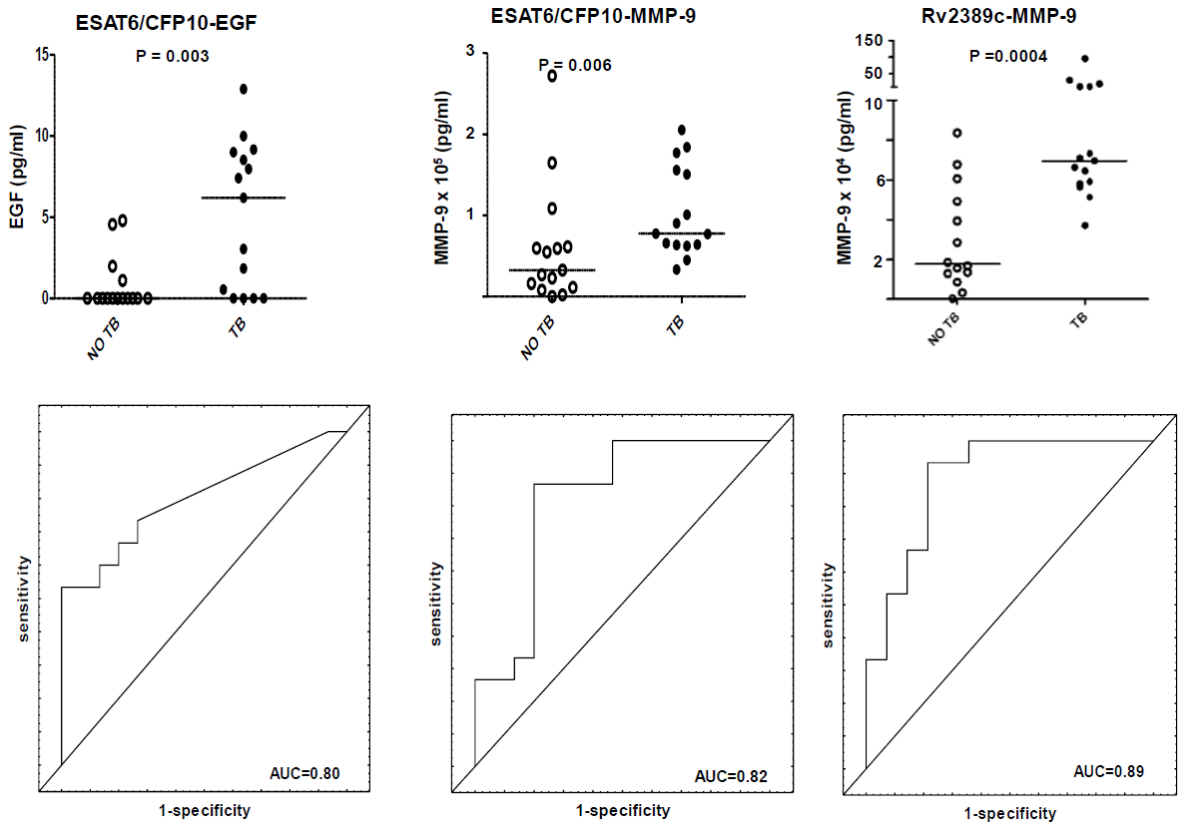
Scatter-dot plots and ROC curves in 7-day WBA. Representative dot plots showing the levels of analytes in the 7-day whole blood culture supernatants and ROC curves for accuracy in the diagnosis of TB disease. Error bars in the dot plots represent the median analyte levels. Representative analytes with AUC≥0.80 are shown.

When the antigen-specific responses were calculated by subtraction of the unstimulated levels from those obtained after stimulation with the respective antigens, ESAT-6/CFP-10-specific levels of EGF, IP-10 and MMP-9 were significantly higher (p<0.05) in TB cases (table 2, figure 1). Following stimulation with Rv2029c, only MMP-9 responses were significantly different between the TB and no TB cases (higher in the TB cases). Similarly, Rv2389c elicited the production of higher levels of MMP-9 and EGF in the TB cases. Only EGF levels were significantly different between the TB and non TB cases (higher in TB cases) following stimulation with Rv2032 (table 2).

When the diagnostic accuracy of the markers obtained in supernatants after 7-day stimulation of whole blood with the antigens was evaluated by ROC curve analysis, the area under the ROC curve (AUC) was above 0.70 (an arbitrary AUC cut-off value used in previous studies) [15] [12], for ESAT-6/CFP-10 specific levels of EGF, IP-10 and MMP-9, Rv2029c-specific levels of MMP-9, Rv2389c-specific levels of EGF and MMP-9, Rv2032-specific levels of EGF, as well as the unstimulated levels of fractalkine, IFN-α2, MMP-2 and SAA (table 2). Using the cut-off values derived after ROC analysis, the unstimulated levels of fractalkine and Rv2389c-specific levels of MMP-9 ascertained TB disease with sensitivities of 100% and 93% respectively, but with lower specificities (53% and 79% respectively). ESAT-6/CFP-10 specific MMP-9 was the only marker that ascertained TB disease with both sensitivity and specificity ≥80% (table 2).

**Figure 2:**
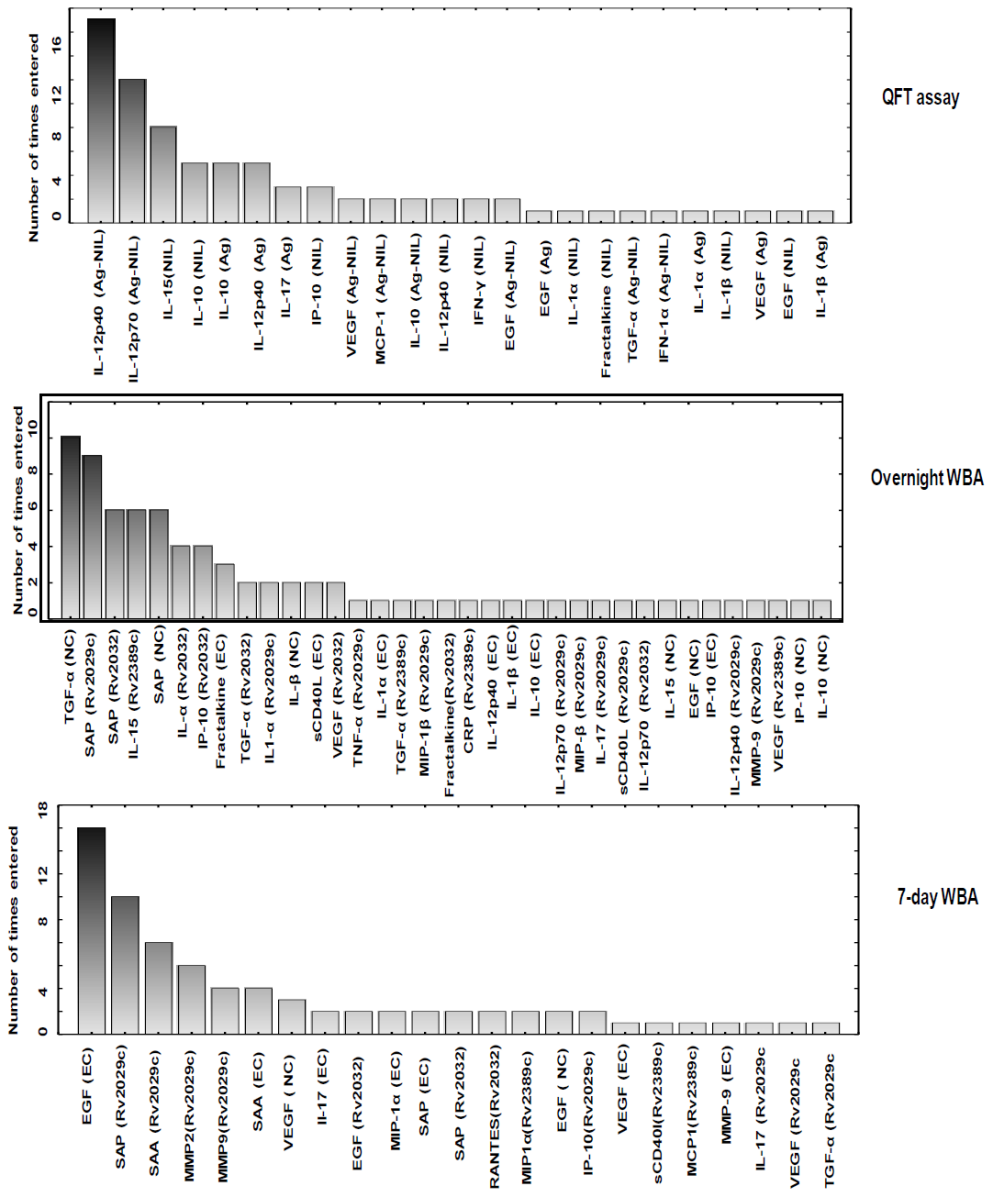
Most frequent analytes in the most accurate GDA models. The columns represent the number of times the analyte was included into the top-20 general discriminant analysis (GDA) models. NC: unstimulated control, Ag: antigen-stimulated marker levels, EC: ESAT-6/CFP-10. QFT = QFT-IT, 1-day = our overnight (in-house) whole blood assay, 7-day = 7-day diluted whole blood culture assay.

When the accuracy of combinations of markers was assessed by general discriminant analysis (GDA), optimal prediction of TB disease was achieved if markers were used in combinations of four. Seven different GDA prediction models accurately classified all the study participants (100%) into their appropriate clinical groups. After leave-one-out cross validation, these models could still correctly classify 100% of the non-TB cases, but only ≤80% of the individuals with active TB (table 3). The most accurate prediction model (ESAT-6/CFP-10-specific EGF, Rv2032-specific VEGF, Rv2029C-specific IL-1beta and Rv2389c-specific MMP-9) accurately classified all participants (100%) after leave-one-out cross validation (table 3). The most frequently occurring analytes in the 20 most accurate predictive models included ESAT-6/CFP-10 specific EGF, Rv2029c-specific SAP and Rv2029c-specific SAA (figure 2).

**Table 3:** Utility of combination of analytes in 7-day culture supernatants in the diagnosis of TB disease.

	Resubstitution Classification Matrix			Leave-one-out Cross validation					
Host marker model (7-day WBA)	% TB cases	% HHCs	Total %	% TB cases	% HHCs	Wilks lambda	f	Error df	P-value
EGF(ESAT6/CFP10), VEGF(Rv2032), MCP-1(Rv2389c), MMP-9(Rv2389c)	93	100	97	93	100	0.862	3.8	24	0.062
EGF(ESAT6/CFP10), VEGF (Rv2032), sCD40L(Rv2389c), MMP-9(Rv2389c)	93	100	97	87	100	0.939	1.6	24	0.22
EGF(ESAT6/CFP10), VEGF (Rv2032), MMP-9(Rv2389c), SAP(Rv2029c)	100	100	100	87	100	0.975	0.57	23	0.459
EGF(ESAT6/CFP10), VEGF(Rv2032), MCP-1(Rv2032), MMP-9(Rv2389c)	93	100	97	93	100	0.866	3.7	24	0.066
EGF(ESAT6/CFP10), VEGF(Rv2032), IP-10(Unstimulated), MMP-9(Rv2389c)	93	100	97	87	100	0.851	4.2	24	0.052
EGF(ESAT6/CFP10), VEGF(Rv2032), Rantes(Unstimulated), MMP-9(Rv2389c)	93	100	97	87	100	0.833	4.8	24	0.038
EGF(ESAT6/CFP10), VEGF(Rv2032), MMP-2(ESAT6/CFP10), MMP-9(Rv2389c)	93	100	97	93	100	0.972	0.7	24	0.420
EGF(ESAT6/CFP10),VEGF(Rv2032) IL-1beta(Rv2029c), MMP-9(Rv2389c)	100	100	100	100	100	0.996	0.1	23	0.768
EGF(ESAT6/CFP10), VEGF(Rv2032), MMP-9(Rv2389c), MCP-1(ESAT6/CFP10)	93	100	97	93	93	0.942	1.5	24	0.239
EGF(ESAT6/CFP10),VEGF(Rv2032),MMP-9(Rv2389c),Fractalkine(Unstimulated)	93	100	97	93	93	0.866	3.7	24	0.066
EGF(ESAT6/CFP10), VEGF(Rv2032), MMP-9(Rv2389c), IL-12p40(Rv2029c)	93	100	97	93	100	0.966	0.8	23	0.384
EGF(ESAT6/CFP10), VEGF(Rv2032), MMP-9(Rv2389c), MIP-1α(Rv2029c)	100	100	100	86	100	0.995	0.1	23	0.757
EGF(ESAT6/CFP10), VEGF (Rv2032), MMP-9(Rv2389c), SAP(Rv2032)	100	100	100	87	100	0.988	0.3	24	0.596
EGF(ESAT6/CFP10), VEGF(Rv2032), MMP-9(Rv2389c), VEGF(Unstimulated)	93	100	97	80	100	0.871	3.4	24	0.072
EGF(ESAT6/CFP10), VEGF(Rv2032), MMP-9(Rv2389c), TNF-α(Rv2029c)	93	100	97	93	100	0.994	0.1	23	0.729
EGF(ESAT6/CFP10), VEGF(Rv2032), MMP-9(Rv2389c), IL-12p40(Rv2032)	100	100	100	80	100	0.980	0.5	24	0.492
EGF(ESAT6/CFP10), VEGF(Rv2032), MMP-9(Rv2389c), CRP(Unstimulated)	93	100	97	80	100	0.913	0.2	24	0.145
EGF(ESAT6/CFP10), VEGF(Rv2032), MMP-9(Rv2389c), EGF(Rv2389c)	93	100	97	80	100	0.912	2.3	24	0.143
EGF(ESAT6/CFP10), VEGF(Rv2032), MMP-9(Rv2389c), MIP-1β(Rv2389c)	100	100	100	93	100	0.998	0.04	23	0.832
EGF(ESAT6/CFP10), VEGF(Rv2032), MMP-9(Rv2389c), IL-1α(Rv2032),	100	100	100	80	100	0.996	0.08	24	0.786

The 4-analyte models were generated by general discriminant analysis. In each case, effect df = 1.

### Utility of host markers detected in overnight culture supernatants (in-house assay) in the diagnosis of TB disease

**Figure 3:**
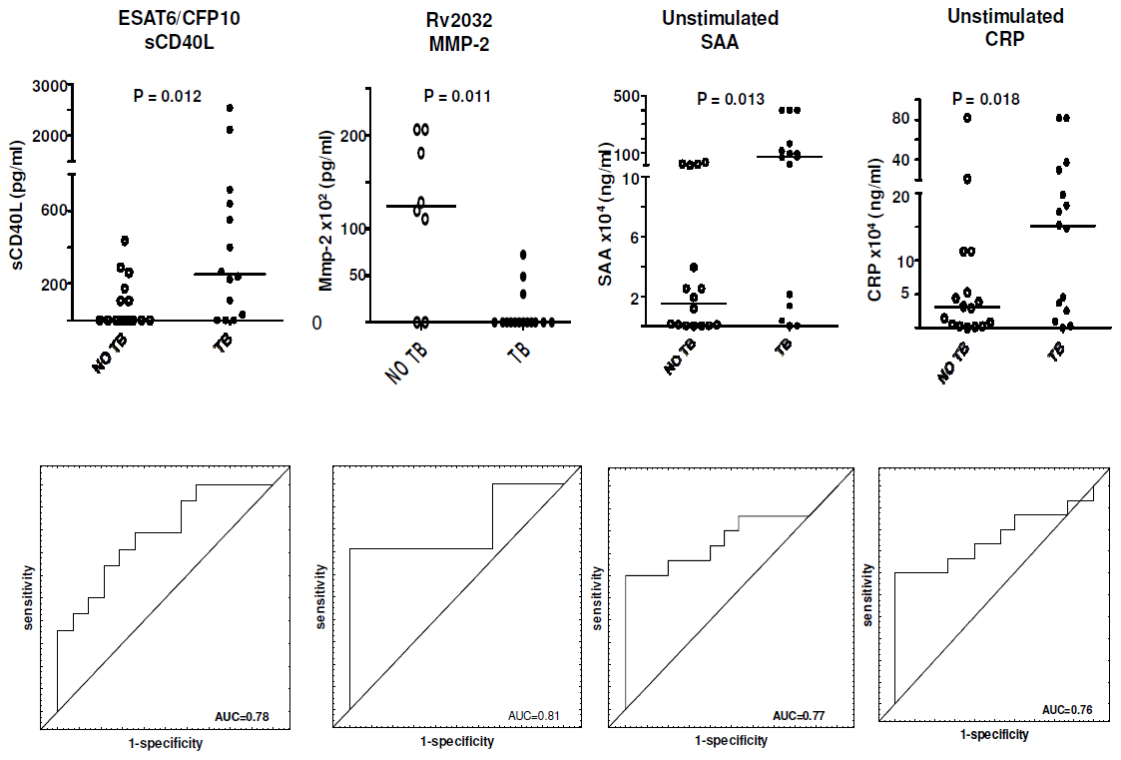
Scatter-dot plots and ROC curves in overnight WBA. Representative plots showing the levels of analytes in the overnight (our *in-house*) whole blood culture supernatants and ROC curves for accuracy in the diagnosis of TB disease. Error bars in the scatter-dot plots represent the median analyte levels. Representative analytes with AUC≥0.76 are shown.

The unstimulated or antigen-specific levels 9 markers (MMP-2, sCD40L, IP-10, IFN-γ, TNF-α, SAA, SAP, and CRP) showed significant differences between the TB cases and controls. Unstimulated CRP and SAA levels were significantly higher in the TB cases. ESAT-6/CFP-10-specific levels of IP-10, sCD40L, TNF-α, IFN-γ and SAP were significantly higher in TB cases. Similarly, Rv2389c-specific levels of SAP and CRP, and Rv2032-specific levels of SAP were higher in the TB cases whereas Rv2032-specific MMP-2 was higher in the non TB cases (table 4, figure 3). When the diagnostic accuracies of the markers detected in overnight culture supernatants were assessed by ROC curve analysis, all the markers that showed significant differences between groups ascertained TB disease with AUC>0.70 (range, 0.71 to 0.83). ESAT-6/CFP-10-specific IP-10 and IFN-γ, Rv2032-specific MMP-2 all diagnosed TB disease with sensitivity ≥93%, but specificity was low for some markers (e.g. 67% for ESAT-6/CFP-10-specific IP-10). By contrast, unstimulated CRP levels ascertained TB with low sensitivity (60%) but with very high specificity (100%) (table 4).

**Table 4:** Diagnostic potential of individual markers detected in in overnight culture supernatants.

Antigen	Marker	TB	No TB	P- value	AUC % (95% CI)	Sensitivity, % (95% CI)	Specificity, % (95% CI)	Cut off value
**ESAT6/ CFP10**	IP-10	16811 (8176–19512)	1826 (0–19353)	0.042	76 (52–93)	93 (66–100)	67 (38–88)	4499.0
**ESAT6/ CFP10**	sCD40L	252 (0–2539)	0 (0–435)	0.012	78 (61–94)	78 (49–95)	60 (32–84)	15.0
**ESAT6/ CFP10**	IFN-γ	339 (8–3578)	8 (0–1558)	0.047	72 (52–92)	93 (66–100)	67 (38–88)	59.8
**ESAT6/ CFP10**	TNF-α	36 (3–169)	3 (0–113)	0.033	0.7 (50–89)	93 (66–100)	53 (27–79)	4.5
**ESAT6/ CFP10**	SAP	4481 (0–22380)	0 (0–17026)	0.025	74(56–93)	86 (57–98)	67 (38–88)	8.7
**Rv2029c**	IP-10	11858 (751–19281)	3474 (0–18433)	0.048	70 (50–89)	67 (34–90)	69 (41–89)	6805.0
**Rv2032**	MMP-2	0 (0–7236)	12379 (0–20606)	0.011	83 (2–100)	100 (78–100)	75 (35–97)	9147.0
**Rv2032**	SAP	5529 (0–24063)	0 (0–20031)	0.023	75 (57–93)	73 (45–92)	73 (45–92)	2842.0
**Rv2389c**	CRP	6317 (0–442665)	12 (0–116862)	0.050	72 (52–92)	69 (39–91)	67 (38–88)	1922.0
**Rv2389c**	SAP	6509.0 (0–22648)	0 (0–10267)	0.032	74 (55–93)	69 (39–91)	60 (32–84)	1216.0
**Unstimulated**	SAA	705597 (560–4000000)	15641 (560–309939)	0.013	76 (58–95)	73 (45–92)	56 (30–80)	20389
**Unstimulated**	CRP	152210 (120–816000)	30463 (170–816000)	0.018	77 (50–89)	60 (32–84)	100 (63–100)	130552

Median levels (and ranges in parenthesis) of analytes detected in overnight (*our in-house*) whole blood culture supernatants and accuracy in the diagnosis of TB disease. All analyte levels are in pg/ml except for CRP, SAA and SAP (ng/ml). P-values were calculated using the Mann Whitney U test. AUC = Area under the receiver operator characteristics curve. 95% CI = 95% confidence interval. The values shown for the the different antigens were corrected for background by subtraction of the unstimulated levels.

When the data obtained from overnight culture supernatants were analysed by the GDA procedure, optimal prediction of TB or no TB disease was achieved if markers were used in combinations of four. The most accurate 4-analyte model (Rv2029c-specific TNF-α+Rv2032-specific IFN-α2+Rv2032-specific SAP+Rv2389c-specific IL-15) accurately classified 92% of the TB cases and 85% of non TB cases after leave-one-out cross validation (table 5). The most frequently occurring analytes in the top 20 predictive models included unstimulated TGF-α and Rv2029c-specific SAP (figure 2).

**Table 5:** Accuracy of combinations of analytes detected in overnight culture supernatants.

	Resubstitution Classification Matrix			Leave-one-out Cross validation					
Host marker model	% TB cases	% HHCs	Total %	% TB cases	% HHCs	Wilks lambda	f	Error df	P-value
TNF-α(Rv2029c), IFN-α2(Rv2032), SAP(Rv2032), IL-15(Rv2389c)	100	85	92	92	85	0.799	5.0	20	0.037
SAP(Rv2029c), TGF-α(Rv2032), SAP(Unstimulated), TGF- α(Unstimulated)	92	79	85	92	64	0.949	1.1	21	0.302
IL-1α(ESAT6/CFP10), SAP(Rv2029c), TGF- α(Rv2389c), SAP(Unstimulated)	100	79	88	92	79	0.909	2.1	21	0.163
fractalkine(ESAT6/CFP10),SAP(Rv2029c), TGF-α(Unstimulated), SAP(Unstimulated)	92	71	80	92	71	0.986	0.3	21	0.602
MIP-1β(Rv2029c), IFN-α2(Rv2032), SAP(Rv2032), IL-15(Rv2389c)	100	85	92	92	77	0.814	4.6	20	0.045
IL-1α(Rv2029c), SAP(Rv2029c), IL-15(Rv2389c), IL-1 α(Unstimulated)	92	71	81	75	64	0.493	21.6	21	0.000
SAP(Rv2029c), fractalkine(Rv2032), TGF- α(Unstimulated), SAP(Unstimulated)	100	86	92	83	71	0.910	2.1	21	0.166
sCD40L(ESAT-6/CFP-10),IL-1α(Rv2029c),TGF-α(Rv2032), CRP(Rv2389c)	92	92	92	92	92	0.623	12.1	20	0.002
IL-12p40(ESAT-6/CFP-10), IP-10(Rv2032), VEGF(Rv2032), TGF-α (Unstimulated)	64	54	59	64	54	0.578	16.0	22	0.000
fractalkine(ESAT-6/CFP-10), IL-1β(ESAT-6/CFP-10), SAP(Rv2029c), TGF-α(Unstimulated)	94	64	77	92	57	0.451	25.5	21	0.000
fractalkine(ESAT-6/CFP-10), IL-10(ESAT-6/CFP-10), SAP(Rv2029c), TGF-α(Unstimulated)	100	64	81	100	57	0.522	19.2	21	0.000
IL-12p70(Rv2029c), IP-10(Rv2032), VEGF(Rv2032), TGF-α (Unstimulated)	92	36	62	58	29	0.632	12.2	21	0.002
MIP-1 α(Rv2029c), IFN- α 2(Rv2032), SAP(Rv2032), IL-15(Rv2389c)	100	77	88	92	77	0.862	3.2	20	0.089
sCD40L(ESAT-6/CFP-10),IL-17(Rv2029c), SAP(Rv2032), IL-15(Rv2389c)	100	85	92	92	85	0.504	19.6	20	0.000
sCD40L(Rv2029c), IL-12p70(Rv2032), SAP(Rv2032), IL-15(Unstimulated)	100	86	92	100	79	0.376	34.8	21	0.000
SAP(Rv2029c), EGF(Unstimulated), TGF- α (Unstimulated), SAP(Unstimulated)	100	67	81	92	67	0.965	0.8	22	0.386
IP-10(ESAT-6/CFP-10), IL-12p40(Rv2029c), IP-10(Rv2032), TGF- α (Unstimulated)	100	85	92	83	85	0.795	5.1	20	0.035
MMP-9(Rv2029c), IFN- α 2(Rv2032), SAP(Rv2032), IL-15(Rv2389c)	92	100	94	92	100	0.752	4.3	13	0.059
IP-10(Rv2032), VEGF(Rv2389c),IP-10(Unstimulated), IL-10(Unstimulated)	77	69	73	62	62	0.825	4.5	21	0.047
SAP(Rv2029c), IL-1 β (Unstimulated), TGF- α (Unstimulated), SAP(Unstimulated)	92	73	91	92	73	0.985	0.3	22	0.570

Accuracy of 4-analyte models generated for markers detected in overnight (*our in-house*) whole blood culture supernatants in the diagnosis of TB disease. In each case, effect df = 1.

### Utility of host markers detected in QFT-IT culture supernatants in the diagnosis of TB disease

**Figure 4:**
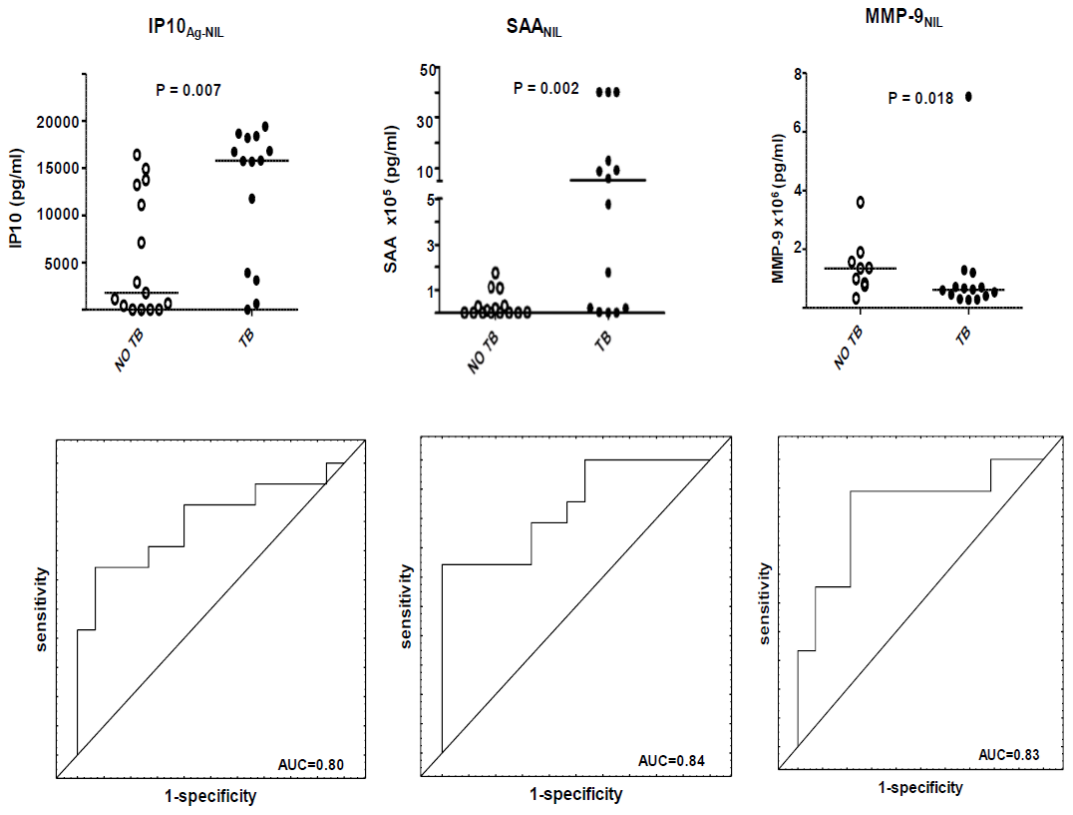
Scatter-dot plots and ROC curves in QFT-IT assay. Representative plots showing the levels of analytes in the QFT-IT whole blood culture supernatants and ROC curves for accuracy in the diagnosis of TB disease. Error bars in the scatter-dot plots represent the median analyte levels. Representative analytes with AUC≥0.82 are shown.

The unstimulated or antigen-specific levels of 11 markers (MCP-1, MIP-1β, VEGF, IP-10, IL-10, IL-1β, TGF-α, CRP, SAA, SAP and MMP-9) showed significant differences when evaluated in QFT-IT supernatants, with most of the discriminatory markers being detected in unstimulated supernatants. The unstimulated levels of 7 markers (MCP-1, MIP-1β, VEGF, IL-10, IL-1β, TGF-α, CRP and MMP-9) were significantly higher in the non-TB cases while the unstimulated levels of CRP, SAA, SAP and IP-10 were significantly higher in the TB cases. The antigen-specific levels of MCP-1 and IP-10 were significantly higher in the TB cases (table 6, figure 4). When the diagnostic accuracy of the data obtained from QFT-IT supernatants was assessed by ROC curve analysis, AUC was ≥0.70 for all the markers showing significant differences between the TB cases and non-cases including unstimulated MCP-1, MIP-1β, VEGF, IL-10, IL-1β, CRP, MMP-9, SAA, CRP and SAP (range 0.72–0.84). When the data was analysed by GDA, eight of the 20 most accurate 4-analyte prediction models accurately classified 100% of the TB cases and up to 89% of the non-TB cases after leave-one-out cross validation (table 7). The most frequently occurring analytes in the top 20 models included the antigen-specific levels of IL-12p70, IL-12p40 and unstimulated IL-15 amongst others (figure 2).

**Table 6:** Diagnostic potential of individual markers detected in QFT-IT supernatants.

Markers	TB cases (n = )	Non TB cases (n = )	P-value	AUC (95% CI)	Sensitivity, % (95% CI)	Specificity, % (95% CI)	Cut off value
**VEGF _Ag_**	100 (2–290)	120 (2–352)	0.085	67 (47–87)	79 (49–95)	60 (32–84)	117.0
**CRP_Ag_**	188210 (3411–816000)	12918 (120–332914)	0.009	80 (62–95)	64 (49–95)	87 (27–78)	72307.0
**SAA_Ag_**	579939 (560–4000000)	1115 (560–222320)	0.004	82 (66–98)	79 (49–95)	73 (45–92)	22752.0
**SAP_Ag_**	48247 (24799–74998)	35242 (560–69282)	0.034	73 (54–93)	79 (49–95)	80 (52–96)	41205.0
**MMP-9_Ag_**	738806 (354239–700362629)	1435154 (278743–2444144)	0.073	72 (49–95)	71 (42–92)	67 (30–93)	935846.0
**IP-10_Ag-Nil_**	15799 (0–19426)	1773 (0–16430)	0.007	80 (65–97)	64 (57–98)	93 (32–84)	15696.0
**MCP-1_Ag-Nil_**	388 (0–7881)	0 (0–12922)	0.047	73 (52–91)	79 (49–95)	67 (38–88)	–732.0
**MCP-1_Nil_**	9969 (1909–18125)	14912 (4368–20001)	0.015	77 (59–94)	60 (66–100)	93 (32–84)	14651.0
**MIP-1β_ Nil_**	1096 (74–2258)	1792 (548–3190)	0.017	76 (59–93)	73 (42–92)	64 (32–84)	1334.0
**TGF-α _Nil_**	8 (2–39)	30 (2–144)	0.047	70 (50–90)	86 (57–98)	60 (32–83)	24.0
**VEGF _Nil_**	31 (2–626)	285 (2–727)	0.041	73 (51–91)	60 (49–95)	79 (32–84)	216.0
**IL-10_ Nil_**	8 (2–51)	41 (2–86)	0.032	74 (55–92)	80 (35–87)	64 (52–96)	11.0
**IL-1β_ Nil_**	108 (2–365)	331 (17–1957)	0.050	72 (53–90)	67 (35–87)	84 (38–88)	254.0
**CRP _Nil_**	457028 (2831–816000)	35080 (157–816000)	0.032	78 (55–92)	64 (42–91)	80 (32–84)	66232.0
**SAA_ Nil_**	1979000 (943–4160000)	1821 (560–173709)	0.002	84 (70–99)	64 (49–95)	100 (38–88)	176199.0
**SAP _Nil_**	44639 (23918–56777)	32733 (22470–79572.)	0.026	76 (55–94)	79 (49–95)	73 (45–92)	38751.0
**MMP-9_ Nil_**	617975 (275049–720100)	1347000 (331645–360500)	0.018	83 (60–100)	89 (49–95)	79 (51–100)	766037.0

Median levels (and ranges in parenthesis) of analytes detected in QFT-IT supernatants and accuracy in the diagnosis of TB disease. All analyte levels are in pg/ml except for CRP, SAA and SAP (ng/ml). P-values were calculated using the Mann Whitney U test. Cut-off values, sensitivity and specificity were selected based on the Youden’s index. AUC = Area under the receiver operator characteristics curve. 95% CI = 95% confidence interval. Nil = unstimulated marker levels, Ag = levels detected in antigen stimulated supernatants, Ag-N = Antigen specific marker levels obtained after subtraction of nil responses.

**Table 7:** Utility of combinations of analytes detected in QFT-IT supernatants in the diagnosis of TB disease.

	Resubstitution Classification Matrix			Leave-one-out Cross validation				
Host marker model	% TB cases	% HHCs	Total %	% TB cases	% HHCs	Wilks lambda	f	P-value
IL-12p40_Ag-Nil,_ IL-12p70_Ag- Nil,_ IL-15_ Nil,_ IL-10_ Nil_	100	100	100	100	89	0.850	3.2	0.092
EGF_Ag_, IL-12p40_Ag-Nil,_ IL-12p70 _Ag- Nil,_ IL-15_ Nil_	100	100	100	100	89	0.844	3.3	0.084
IL-12p40 _Ag-Nil,_ IL-12p70_Ag- Nil,_ IL-15_ Nil,_ IL-1β_ Nil_	100	89	96	100	89	0.601	11.9	0.002
IL-17_Ag_, VEGF_Ag-Nil_, fractalkine _Nil_, IP-10_Nil_	100	100	100	100	89	0.433	23.5	0.000
IL-10_Ag_, IL-12p40 _Ag-Nil,_ IL-12p70 _Ag-Nil,_ TGF-α_ Ag-Nil_	100	89	96	100	89	0.994	0.1	0.745
IL-17_Ag_, MCP-1_ Ag-Nil,_ VEGF_ Ag-Nil ,_IP-10 _Nil_	100	100	100	100	100	0.426	24.2	0.000
IL-12p40 _Ag_, IL-10 _Ag_, IL-10_ Ag-Nil,_ IL-12p70 _Ag- Nil_	100	100	100	100	89	0.812	4.2	0.056
IL-12p40 _Ag,_ IL-12p40 _Nil,_ IL-12p70 _Ag- Nil,_ IFN-γ_ Nil_	93	100	96	93	89	0.886	2.3	0.147
IL-12p40 _Ag_, IL-10 _Ag_IL-12p70 _Ag- Nil,_ IL-10 _Nil_	100	100	100	100	89	0.794	4.7	0.045
IL-10_Ag_, EGF_ Ag- Nil_IFN-α2_ Ag- Nil,_ IL-12p70 _Ag- Nil_	93	100	96	93	89	0.890	2.2	0.152
IL-12p40 _Ag_, IL-12p40 _Ag-Nil_ IL-12p70 _Ag- Nil_, IL-10_Nil_	100	100	100	93	100	0.929	1.4	0.256
IL-1α_Ag_, IL-12p40_Ag-Nil,_ IL-12p70 _Ag- Nil,_ IL-15_ Nil_	100	89	96	100	89	0.571	13.5	0.002
IL-1α_Nil_, IL-12p40_Ag-Nil,_ IL-12p70 _Ag- Nil,_ IL-15_ Nil_	100	100	100	100	89	0.667	9.0	0.008
IL-17Ag, EGF_ Ag-Nil,_ IL-12p40 _Nil_, IP-10_Nil_	93	100	96	93	89	0.371	30.5	0.000
VEGF_Ag_, IL-12p40_Ag-Nil,_ IL-12p70 _Ag- Nil_, IL-15_ Nil_	100	89	96	100	89	0.661	9.2	0.007
IL-12p40 _Ag-Nil,_ IL-12p70 _Ag- Nil,_ IL-10 _Nil_, IFN-γ _Nil_	93	100	96	93	89	0.921	1.5	0.230
IL-10_Ag_, MCP-1_ Ag- Nil,_ IL-12p40_Ag-Nil,_ IL-12p70_Ag- Nil_	100	89	96	93	89	0.995	0.1	0.768
IL-12p40_Ag-Nil,_ IL-12p70_Ag- Nil_ EGF _Nil_, IL-15_Nil_	100	100	100	100	78	0.886	2.3	0.147
IL-12p40_Ag-Nil,_ IL-12p70_Ag- Nil_ IL-1β_Ag_, IL-15_Nil_	100	100	100	100	89	0.760	5.7	0.028
IL-12p40 _Ag,_ IL-12p70 _Ag- Nil_ IL-10_Nil_, IL-10_ Ag- Nil_	100	100	100	100	89	0.786	4.9	0.040

Accuracy of 4-analyte GDA models in QFT-IT supernatants in the diagnosis of TB disease. Nil = unstimulated marker levels, Ag = levels detected in antigen stimulated supernatants, Ag-N = Antigen-specific biomarker levels obtained after subtraction of Nil responses. In each case, effect df = 1, error df = 18.

### Correlation between host marker levels detected in the overnight, 7-day and QFT-IT whole blood culture assays

The marker levels obtained after stimulation with each antigen (Rv2029c, Rv2032, Rv2389c and ESAT-6/CFP-10) or the unstimulated control were compared between the assays (QFT-IT, the in-house overnight WBA and the in-house 7-day WBA). Comparison between the assay types was only possible for ESAT-6/CFP-10-specific and the unstimulated control responses as these were the only common conditions between all the assay types. Because of the relatively large number of markers evaluated in this study, only analytes that showed significant differences between the TB cases and non-cases in at least one of the assay types were included in the analysis.

Of the 26 analytes evaluated, 12 (fractalkine, IFN-α2, MCP-1, MIP-1β, VEGF, IL-1β, IL-10, MMP-2, MMP-9, CRP, SAA, and SAP) showed significant differences between the TB cases and non cases in at least one of the three assays, when evaluated in unstimulated supernatants. The levels of the markers were compared across assays. The unstimulated levels of MCP-1, MIP-1β and IL-1β were significantly higher in QFT-IT supernatants compared to the two in-house WBAs (table 8). QFT-IT levels of the remaining unstimulated markers were similar to the levels obtained in our in-house WBA. With the exception of fractalkine, all unstimulated analyte levels were significantly lower (2–200 fold) in the 7-day WBA supernatants in comparison to either the QFT-IT or the in-house overnight WBA.

**Table 8:** Correlation between unstimulated and ESAT-6/CFP-10-specific analyte levels detected in supernatants from the three culture techniques.

	QFT-IT Vs overnight		QFT-IT vs 7-day		Overnight vs 7 day		Fold difference	
	r^2^	P value	r^2^	P value	r^2^	P value	QFT vs overnight	QFT vs 7-day WBA
ESAT6/CFP10 IP10	**0.63**	**0.0004**	**0.60**	**0.001**	**0.78**	**< 0.0001**	1.3	4.4
ESAT6/CFP10 MCP-1	0.53	0.004	0.35	0.076	0.20	0.31	0.0005	0.0001
ESAT6/CFP10 IFN-γ	0.85	1.44	0.84	3.96	**0.77**	**< 0.0001**	2.5	179
ESAT6/CFP10 EGF	−0.16	0.41	−0.20	0.31	0.29	0.15	1	1
ESAT6/CFP10 MMP-9	**0.62**	**0.002**	0.19	0.40	0.19	0.40	1.4	1.3
ESAT6/CFP10 sCD40L	0.05	0.78	−0.15	0.44	−0.01	0.97	8.1	39.1
ESAT6/CFP10 SAP	**0.74**	**< 0.0001**	0.06	0.75	0.15	0.43	0.75	15.7
Unstimulated Fractalkine	0.40	0.031	0.42	0.021	016	0.41	0.5	1.1
Unstimulated IFN-α2	**0.98**	**< 0.0001**	0.18	0.35	0.16	0.40	2	5.7
UnstimulatedMCP-1	0.07	0.73	−0.07	0.72	0.14	0.47	15	200
UnstimulatedMIP-1β	0.45	0.015	0.17	0.39	0.41	0.03	109	746
UnstimulatedVEGF	0.32	0.096	0.08	0.68	0.26	0.17	1.3	4.7
UnstimulatedIL-1β	**0.65**	**0.0001**	0.12	0.55	0.40	0.03	115	115
Unstimulated IL-10	**0.63**	**0.0003**	−0.18	0.36	−0.15	0.43	2	5.7
Unstimulated MMP-2	**0.73**	**0.0001**	0.41	0.06	0.07	0.76	1	23
Unstimulated MMP-9	**0.62**	**0.002**	0.19	0.40	0.19	0.41	2.4	13.5
Unstimulated CRP	**0.95**	**< 0.0001**	0.38	0.044	0.40	0.033	1.1	39.8
Unstimulated SAA	**1.0**	**< 0.0001**	**0.91**	**< 0.0001**	**0.90**	**< 0.0001**	0.7	39
Unstimulated SAP	**0.74**	**< 0.0001**	0.06	0.75	0.15	0.43	1.2	36.3

Correlation matrices were only computed for analytes that discriminated between TB and no-TB with AUC>0.70 in at least one of the WBA techniques. Analytes with r^2^ values >0.5 and p values <0.01 are highlighted bold. The mean values of the markers in the different culture techniques were compared.

Fourteen ESAT-6/CFP-10-induced markers could discriminate between study groups (TB vs. no TB) in at least one of the assays. There were generally no significant differences in the levels of markers upon stimulation with ESAT-6/CFP-10 between the QFT-IT and our in-house overnight WBA, but generally lower levels were obtained in the 7-day WBA supernatants (table 8).

**Figure 5:**
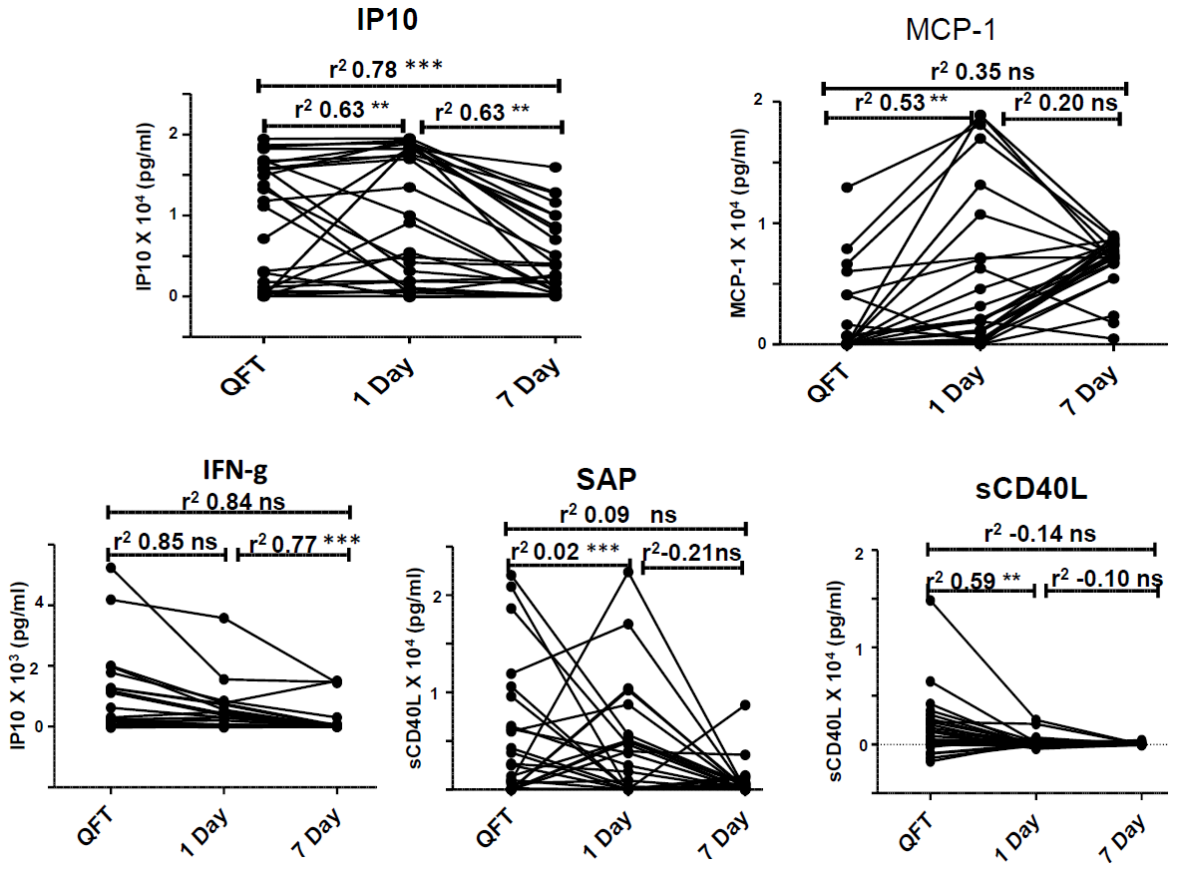
Correlation of stimulated analyte levels across assays. Correlation between ESAT-6/CFP-10-stimulated analyte levels in supernatants from the three whole blood cultures. Representative plots for 5 ESAT-6/CFP-10-induced host markers are shown. ns = non-significant, * = P value<0.05, ** = P value ≤0.001, *** = P value<0.0001.

**Figure 6:**
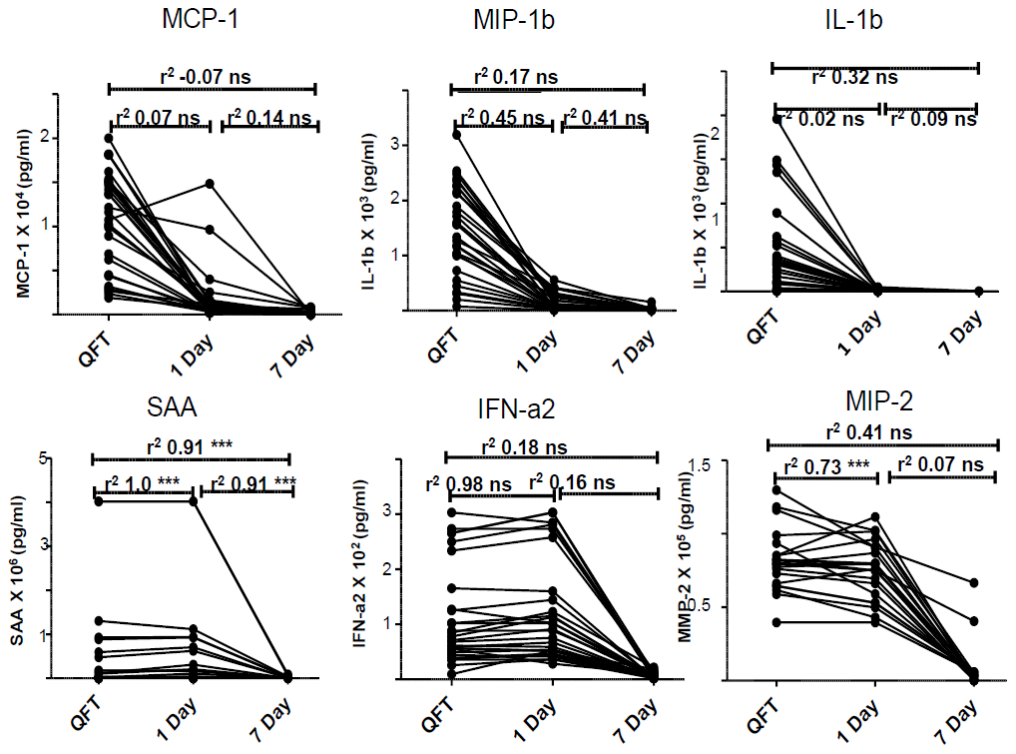
Correlation of unstimulated analyte levels across assays. Correlation between analytes detected in unstimulated supernatants in the three whole blood culture techniques. Representative plots for 6 host markers are shown. ns = non-significant, * = P value<0.05, ** = P value ≤0.001, *** = P value<0.0001.

ESAT-6/CFP10-specific IP-10 and unstimulated SAA were the only markers that discriminated between the TB cases and non-cases in all three WBA types (tables 2, 4 and 6). The levels of the two analytes also correlated positively between all the three assay types (r^2^ = 1, p< 0.0001 in some cases). The highest significant correlations were observed between the QFT-IT and our in-house overnight WBA responses (table 8, figures 5 and 6).

## Discussion

In this study the potential of host markers detectable in supernatants of *M.tb* infection phase-dependent antigen stimulated whole blood culture assays was investigated in an overnight and 7-day stimulation format for the diagnosis of TB disease. Our data indicates that the *M.tb* antigens evaluated in this study elicit a variety of host responses after short-term incubation with whole blood. The most promising single markers obtained after overnight culture included the Rv2032-specific levels of MMP-2, ESAT-6/CFP10-induced sCD40L and IP-10 and unstimulated CRP and SAA levels. Biosignatures comprising different four-analyte combinations showed promise.

We previously showed that stimulation of whole blood with novel *M.tb* infection phase dependent antigens including Rv2032, Rv0081, Rv1737c, Rv0867c and Rv2389c resulted in the production of multiple host markers, some of which showed potential in the diagnosis of TB disease with high accuracy [15]. Because the 7-day WBA employed in our previous studies [15], [18] would not be optimal as a diagnostic method for TB disease in operational terms, we investigated if the antigens also had the potential to elicit the production of host markers in short-term (overnight) cultured assays. The potential clinical usefulness of any promising diagnostic candidate markers obtained in the short-term assay would be evaluated prospectively in future larger validation studies.

In the current pilot study, we enrolled 15 pulmonary TB cases and 15 individuals that presented at the health care facility with symptoms requiring investigation for TB, but in whom TB disease was finally ruled out. In our investigation of candidate diagnostic markers, we compared the levels of markers in the individuals with confirmed TB disease to all those in whom TB disease was ruled out (50% of whom were QFT-IT positive), regardless of QFT-IT status. Although it is important to evaluate which markers show differences between different *M.tb* infection states, for example, comparing individuals with active TB disease vs. completely uninfected (QFT-IT negative) individuals, or active disease vs. latently infected (QFT-IT positive) individuals, we only focused on the differences between TB disease and no TB disease because the aim of this pilot study and that of the larger ongoing project, was to identify markers that would be suitable for use in the diagnosis of active TB, even in high burden settings, where the prevalence of LTBI is high.

Rv2032 specific MMP-2 was the most promising antigen-stimulated single marker obtained in the short-term WBA experiment, with an AUC of 83%, whereas the antigen-specific levels of EGF, MMP-9 and IP-10 amongst others, and the unstimulated levels of CRP, SAA and SAP also showed potential especially when the 7-day WBA and QFT-IT supernatant results were taken into account (tables 4 and 6). However, no individual marker consistently showed potential in all the three different WBA types, with ESAT-6/CFP-10 specific IP-10 and unstimulated SAA being the only markers showing differences between groups in all the three WBAs. As observed in our previous studies [15] the predictive abilities of markers improved when analytes were used in combinations of four. The acute phase proteins (CRP, SAA) alongside other host markers including TGF-α, EGF, VEGF, IFN-α2 featured strongly in the top four-analyte multi-marker models obtained in the different WBAs. The inclusion of TGF-α, EGF, VEGF and IFN-α2 into the best discriminatory marker models is consistent with findings from previous studies conducted on participants from the same community [12] [15]. Consistent with these previous observations, the unstimulated levels of IFN-α2 were higher in non-TB cases than in TB cases [19]. The reason for the higher levels of some markers in non-TB cases is not clear but could be due to other respiratory conditions in the non-TB cases, given that the individuals included in this and our previous study as controls actually presented at the health care centre with symptoms suggestive of respiratory infections and hence, were investigated for TB. Although we have not previously evaluated the acute phase proteins (CRP and SAA) in previous antigen-stimulated experiments on participants from the same community, the inclusion of especially the unstimulated levels of these markers into the top models is not surprising given that serum levels of these markers have previously been shown to be potentially useful in the diagnosis of TB disease [20]. Although the inclusion of these markers into the most promising discriminatory marker models implies that the markers were useful in this small study, it should be noted that the focus of the current small study was not on identifying a specific combination of host markers as the “best diagnostic model” for TB disease, but on the identification of markers which could be evaluated further in larger prospective studies. Therefore, all the markers that were most frequently included in the multi-marker models as shown in figure 2 deserve further evaluation in future studies.

MMPs and acute phase proteins were amongst the most important markers identified in this small study. These markers have been shown to be involved in many diseases. Increased levels of MMP-2 and MMP-9 have been previously associated with lung and breast cancers [21] [22] [23]. CRP and SAA are widely used as biomarkers for a number of conditions including pulmonary infections [24] [25]. Although serum levels of CRP and SAA have previously been shown to be potentially useful in the diagnosis of TB disease [26] [20], no TB diagnostic test employing these markers currently exists. Both the MMPs and the acute phase proteins occurred frequently in the top four-analyte models that most accurately discriminated between TB disease and no TB, but none of these markers was consistently included in the top models in all the WBA types. The results of this small study should therefore be interpreted with caution, given the limited numbers of study participants. That notwithstanding, if the markers identified in this study are consistently shown to be important in the diagnosis of TB disease either individually or in combination with other markers in future larger studies, the detection MMPs and acute phase proteins in clinical samples will be easier, given the very high levels obtained in all study participants. This is in contrast to markers such as EGF and TGF-α which also featured in top marker models in previous studies conducted in the same study community [19] [12], but which are expressed at low levels.

In addition to the classical TB antigens (ESAT-6 and CFP-10), evaluated in this study as a fusion protein, three other relatively new recombinant antigens (Rv2029c, Rv2032 and Rv2389c) were evaluated in the overnight and 7-day WBAs. As previously discussed [12], Rv2029c and Rv2032 are DosR regulon encoded antigens, whereas Rv2389c is a resuscitation promoting factor (RPF). The DosR regulon of *M.tb* is upregulated when *M.tb* is subjected to conditions that mimic latency including hypoxia, nutrient starvation, low nitric oxide or low pH [27] [28]. Rpfs are growth-promoting proteins and have been shown to resuscitate dormant bacteria, as well as enhance the growth of bacteria, including *M.tb* in both liquid and solid media [29]. All the antigens evaluated in the current study elicited host responses in both TB and non-TB cases. This is not surprising given that 50% of our non-TB cases were infected with *M.tb*, according to the QFT-IT test [15] [12] [30] [18]. Furthermore, it is known that *M.tb* infection is a spectrum [31] [32]. Higher concentrations of some markers (for example, MMP-9 responses to Rv2029c and Rv2389c in this study) in TB cases might therefore be expected as previously discussed in [12].

Three of the antigens evaluated in this pilot study (Rv2032, Rv2389c and ESAT-6/CFP-10) were previously evaluated in our initial study evaluating the levels of multiple host markers in novel *M.tb* infection phase-dependent antigens in 7-day WBA supernatants [15]. The promise shown by ESAT-6/CFP-10 specific EGF in this study is consistent with the findings of the previous study. However, the potential shown ESAT-6/CFP-10 specific TGF-α and TNF-α were not replicated in 7-day WBA supernatants in this study. Similarly, differences observed between the TB cases and non-cases for Rv2389c- specific levels of TGF-α, TNF-α, VEGF, IL-10 ,and Rv2032-specific levels of fractalkine, IL-12(p40), TGF-α, TNF-α, VEGF and IL-10 were not replicated in the 7-day WBA supernatants in the current study. The reason for the discrepancies between the findings of the initial study and the current 7-day WBA results is unknown but may relate to the small sample size employed in the current study and differences in the antibodies employed in the two studies. The recurrence of common antigen-specific markers, for example, Rv2032-specific VEGF in diagnostic models in both studies indicates the results between the two studies might be identical, if very similar conditions were employed. However, the current ongoing larger validation studies in different African countries will help identify the best candidate diagnostic markers and models.

Although the WBAs employed in the current study are different (7-day culture vs. overnight culture, and 5-fold dilution of whole blood in the 7-day WBA vs. undiluted blood in the overnight assay), we compared the levels of host markers detected in supernatants from each study participant after culture using the different techniques. This was done in order to ascertain if any of the potentially useful markers performs consistently in the different WBA types. Furthermore, we assessed the correlation between our in-house overnight WBA and the highly standardised QFT-IT assay. The generally good correlation between the markers detected in culture supernatants from our in-house overnight WBA with those detected in the QFT-IT supernatants is reassuring and suggests that both assay types are suitable for further diagnostic marker discovery. The magnitude of responses obtained with the two overnight assays was consistently higher than the responses obtained in the 7-day WBAs. This could be explained by the amount of sample that was used in the different assay types. The two overnight assays (our in-house WBA and the QFT-IT assay) employed 1 ml of undiluted whole blood while blood samples were diluted five times before being evaluated in the 7-day WBA as previously recommended [12, [33]. A direct comparison of the levels of markers detected using the three assay types was only possible for the unstimulated and ESAT-6/CFP-10-stimulated analyte levels (although the QFT-IT assay employs a third antigen (TB7.7) in addition to ESAT-6/CFP-10), since these were the only common conditions across the WBA techniques. Higher responses were observed in the QFT-IT antigen-stimulated and unstimulated supernatants for some markers, in comparison to the levels obtained in our in-house WBA supernatants. The reasons for this observation are unclear as the same amount of undiluted blood was evaluated using the two culture techniques. However, the QFT-IT system is a highly validated and standardized test system and uses closed tubes and blood was drawn directly into the tubes. In comparison, blood was drawn into sodium heparinized tubes before being aliquoted into the wells of 24-well tissue culture plates in our in-house assay. This observation was not investigated further as the aim of the current study was to investigate if the relatively newer antigens elicited the production of host markers after overnight stimulation, as opposed to the traditional 7-day cultures, and whether the markers possessed diagnostic potential for TB disease.

IGRAs are well established and remain the tests of choice for the diagnosis of LTBI in many settings. However, these assays do not discriminate between LTBI and active disease and are therefore not recommended in high burden settings [34]. This is mainly because of the large proportion of LTBI cases in high-burden settings. The current study was performed in a setting with high burden of TB. The results of the current as well as those obtained in our previous studies [15] raise the hope that any diagnostic tests based on these antigens and host markers, might be highly suitable in high-burden settings. However, diagnostic tests based on the antigens and host markers would be mostly beneficial to TB control programs if such antigens and markers are incorporated into rapid, point-of-care test platforms such as the lateral flow technology. Such test platforms (based on the Up-converting phosphor imaging technology), are currently being investigated for many infectious diseases including TB [35] [36], and have the ability to detect multiple host markers on a single strip. Although a diagnostic modality based on overnight stimulation of whole blood with *M.tb* specific antigens will only yield results within 24 hours after the patient’s visit as previously discussed [15], it might prove cheaper and more suitable in remote settings than the GeneXpert and conventional *M.tb* culture. The latter has been the gold standard for a long time, but is not widely available in the most highly burdened TB and resource-constrained settings, and also dependent on the presence of sufficient *M.tb* organisms in sputum, which may be hard to obtain reliably, particularly in the context of HIV. A non-sputum based test will also be very useful for other individuals with difficulty in providing good quality sputum samples, such as those with extra-pulmonary TB and children.

In addition to the small sample size, another limitation of our study is the case-control design. However, such a design was the most appropriate for the current study as this was a pilot study for a larger trial that is ongoing in seven African field sites with five European partners. Larger studies, especially studies employing short term culture assays are needed to validate the findings of this study, and to further down-select and validate the most accurate 3 to 5 antigen-specific or unstimulated (ex vivo) host markers. Results of such studies will be highly beneficial to the field if HIV infected individuals, those with extrapulmonary TB disease and those with other lung infections, including pneumonia are included and such studies should ideally be prospective studies of participants with suspected TB. Any validated host markers could then be incorporated into lateral flow devices.

## Conclusion

In conclusion, the results of the current study indicate that novel *M.tb* infection phase dependent antigens including Rv2029c, Rv2032 and Rv2389c elicit the production of several host markers, some of which have potential to be useful as TB diagnostic candidates either singly or in combinations, in overnight whole blood culture assays. Although none of the single antigen-induced host markers ascertained TB disease with an AUC >0.85, overnight culture assays yielded several four-marker models with more than 85% sensitivity and specificity in this small study. The potentially useful analytes identified in this pilot study require further investigation in larger studies.
